# Oxidized LDL, homocysteine, homocysteine thiolactone and advanced glycation end products act as pro-oxidant metabolites inducing cytokine release, macrophage infiltration and pro-angiogenic effect in ARPE-19 cells

**DOI:** 10.1371/journal.pone.0216899

**Published:** 2019-05-14

**Authors:** Kannadasan AnandBabu, Parveen Sen, Narayanasamy Angayarkanni

**Affiliations:** 1 R.S. Mehta Jain Department of Biochemistry and Cell Biology, KBIRVO, Vision Research Foundation, Sankara Nethralaya, Chennai, India; 2 School of Chemical and Biotechnology, SASTRA University, Thanjavur, India; 3 Shri Bhagwan Mahavir Vitreoretinal Services, Sankara Nethralaya, Chennai, India; University of Florida, UNITED STATES

## Abstract

Age-related Macular Degeneration (AMD) is one of the major vision-threatening diseases of the eye. Oxidative stress is one of the key factors in the onset and progression of AMD. In this study, metabolites associated with AMD pathology more so at the systemic level namely, oxidized LDL (oxLDL), homocysteine (Hcy), homocysteine thiolactone (HCTL), advanced glycation end product (AGE) were evaluated for their pro-oxidant nature in a localized ocular environment based on *in vitro* studies in human retinal pigment epithelial cells (ARPE-19 cells). Human ARPE-19 cells were treated with pro-oxidants 50 μg/mL oxLDL, 500 μM Hcy, 500 nM HCTL, 100 μg/mL AGE, 200 μM H_2_O_2_ and 200 μM H_2_O_2_ with and without pre-treatment of 5 mM N-acetyl cysteine (NAC). The cytokines IL-6, IL-8 and vascular endothelial growth factor (VEGF) secreted from ARPE-19 cells exposed to pro-oxidants were estimated by ELISA. *In vitro* angiogenesis assay was performed with conditioned media of the pro-oxidant treated ARPE-19 cells in Geltrex-Matrigel coated 96-well plate. The human acute monocytic leukemia cell line (THP-1) was differentiated into macrophages and its migration in response to conditioned media of ARPE-19 cells insulted with the pro-oxidants was studied by transwell migration assay. Western blot was performed to detect the protein expression of Bax, Bcl-2 and NF-κB to assess apoptotic changes. The compounds involved in the study showed a significant increase in reactive oxygen species (ROS) generation in ARPE-19 cells (oxLDL; Hcy; AGE: *p < 0*.*001* and HCTL: *p < 0*.*05*). NAC pre-treatment significantly lowered the oxidative stress brought about by pro-oxidants as seen by lowered ROS and MDA levels in the cells. Treatment with pro-oxidants significantly increased the secretion of IL-6 (oxLDL: *p < 0*.*05*; Hcy, HCTL and AGE: *p < 0*.*01*) and IL-8 cytokines (oxLDL: *p < 0*.*05*; HCTL: *p <*. *001* and AGE: *p < 0*.*01*) in ARPE-19 cells. Serum samples of AMD patients (n = 23) revealed significantly higher IL-6 and IL-8 levels compared to control subjects (n = 23) (IL6: *p < 0*.*01* and IL8: *p < 0*.*05*). The pro-oxidants also promoted VEGF secretion by ARPE-19 cells compared to untreated control (oxLDL: *p < 0*.*001*; Hcy: *p < 0*.*01*; HCTL and AGE: *p < 0*.*05*). *In vitro* angiogenesis assay showed that the conditioned media significantly increased the tube formation in RF/6A endothelial cells. Transwell migration assay revealed significant infiltration of macrophages in response to pro-oxidants. We further demonstrated that the pro-oxidants increased the Bax/Bcl-2 ratio and increased the NF-κB activation resulting in pro-apoptotic changes in ARPE-19 cells. Thus, oxLDL, Hcy, HCTL and AGE act as pro-oxidant metabolites in RPE that promote AMD through oxidative stress, inflammation, chemotaxis and neovascularization.

## Introduction

Age-related macular degeneration (AMD) is a multifactorial disease, characterized by degeneration of retinal pigment epithelium (RPE) and photoreceptors in the macula. It is the leading cause of blindness in the elderly in many developed countries [1]. The retina and RPE are highly exposed to oxidative stress conditions due to intense light, increased lipofuscin formation as well as hypoxia, all of which contribute to the generation of reactive oxygen species (ROS) thereby promoting AMD pathogenesis in the early stage of the disease [2]. Oxidative stress, deranged lipid metabolism and inflammation play a major role in the pathogenesis of AMD [3–5].

Oxidation of LDL is a key atherogenic phenomenon in cardiovascular diseases (CVD) [6]. There are shared risk factors and pathogenic mechanisms in AMD and CVD, though the association between the two has not been clearly established [7]. This study explored these pro-oxidant factors associated with LDL modification in the context of RPE dysfunction relevant to AMD.

Elevated concentrations of plasma oxidized low-density lipoprotein (oxLDL) is one of the risk factors for AMD [8]. Our previous study reported on the elevated serum oxLDL in AMD [9]. OxLDL is a known atherogenic metabolite that is pro-inflammatory in nature [10]. However, there are limited studies on the role of oxLDL in AMD pathology not only at the systemic level but also at the level of RPE in the eye. Picard et al reported on the sub-RPE accumulation of oxLDL along with basement membrane thickening associated with AMD pathology [11].

Several metabolites are associated with LDL oxidation. Elevated plasma homocysteine (Hcy) as well as homocysteine thiolactone (HCTL), are metabolites associated with the AMD pathology [12–14] as excess homocysteine affects the RPE structure and function [15]. Hcy brings about LDL oxidation through modification of LDL apoB [16].

The advanced glycation end product (AGE), a key pathophysiological metabolite is associated with cardiovascular diseases [17,18], stroke [19] and AMD [20]. The AGE levels associated with aging contributes to RPE dysfunction in the pathogenesis of choroidal neovascularization (CNV) in AMD [21–23]. AGE can also form modified LDL [24].

The aim of the study is to see if the pathogenic molecules associated with AMD namely oxLDL, Hcy, HCTL and AGE cause pro-oxidant, pro-inflammatory and pro-angiogenic responses in the local environment of RPE as evaluated in ARPE-19 cells *in vitro*.

## Materials and methods

### ARPE-19 culture

The human Retinal Pigment Epithelial cells, ARPE-19 (ATCC-CRL-2302) was cultured using Dulbecco’s Modified Eagle medium/Ham’s F12 medium (DMEM/F-12; Sigma-Aldrich, USA) supplemented with 10% Fetal Bovine Serum (FBS; Gibco, USA), Antibiotic-Antimycotic solution (Gibco, USA). At 80% confluency, they were used for the experiments in DMEM/F-12 supplemented with 1% FBS (low serum media). The cells were then exposed to 50 μg/mL oxLDL, 50 μg/mL Native LDL (N.LDL), 500 μM Hcy (Sigma-Aldrich, USA), 500 nM HCTL (Sigma-Aldrich, USA), 100 μg/mL AGE, 200 μM H_2_O_2_ (Merck, USA) and pre-treatment with 5 mM N-acetylcysteine (NAC; Sigma-Aldrich, USA).

### THP-1 Macrophage culture

The human acute monocytic leukemia cell line, THP-1 (Riken, Japan) was differentiated into macrophages using 50 ng/mL phorbol 12-myristate-13-acetate (PMA; Sigma-Aldrich, USA) for 48 h with Roswell Park Memorial Institute 1640 medium (RPMI 1640; Biowest, France), supplemented with 10% FBS. After 48 h, they were subjected to the experimental conditions.

### LDL isolation by density gradient method

LDL was isolated from human plasma by density gradient method using Optiprep (60% Iodixanol solution) (Axis-Shield, Norway) by the method of Davies et al [25]. Human plasma was mixed with Optiprep to obtain 12% iodixanol as a working solution. Optiprep was mixed with PBS to arrive at 9% Iodixanol solution which was dispensed into Optiseal centrifuge tubes (Beckman Coulter, USA) and 3 mL of the iodixanol working solution was carefully under-layered with a syringe and metal cannula. The tubes were placed in NVT 65 near-vertical rotor (Beckman Coulter, USA) and centrifuged at 60,000 rpm for 3 h at 16°C in a Beckman Optima XL-100 ultracentrifuge (Beckman Coulter, USA). LDL separated as interphase was aspirated with syringe and metal cannula and concentrated using SpeedVac Concentrator (Thermo Fisher Scientific, USA).

### *In vitro* oxidation of LDL and validation of LDL oxidation

The oxLDL was generated by incubating the plasma LDL with 10 μM CuCl_2_ at 37°C for 24 h [26]. Oxidation of LDL was confirmed based on the shift in absorbance at 234 nm [27]. The A_234_ before and after oxidation was 0.3802 and 0.5442, respectively. In addition, peroxidation of LDL was measured by the determination of thiobarbituric acid-reactive substances (TBARS) and expressed as malondialdehyde (MDA) equivalents [28]. Native LDL (N.LDL) and the corresponding oxLDL showed MDA levels of 3.76 ± 1.86 and 65.97 ± 6.26, respectively showing oxidation of LDL. Reactive free amino groups in N.LDL and oxLDL were estimated using spectrophotometer (Beckman Coulter, USA) by the method of Urs P. Steinbrecher [29]. Accordingly, there was a 30% decrease in the reactive amino group in oxLDL compared to N.LDL indicating LDL oxidation.

### Measurement of intracellular reactive oxygen species

ARPE-19 cells were plated in a 96-well plate at a density of 1 x 10^4^ cells/well. The cells were washed with PBS and 10 μM 2’-7” dichlorofluorescein diacetate (DCFH-DA) (Sigma-Aldrich, USA) in serum-free media was added to each well and incubated for 30 min at 37°C. Excess unused DCFH-DA was removed, washed once with PBS followed by incubation with the compounds of interest and the fluorescence was measured using SpectraMax M2e (Molecular Devices, USA) at Ex485 nm and Em530 nm in kinetic mode for one hour. ROS production was expressed as relative fluorescence. NAC (5 mM) was used as antioxidant, while H_2_O_2_ (200 μM) was used as the pro-oxidant (positive control). The cell homogenates were used in the MDA assay according to the method of Yokode et al., [28] to assess the intracellular oxidative stress.

### MTT assay for cell viability

Cells were plated in a 96-well plate at a density of 1 x 10^4^ cells/well. After exposing the cells to the compound of interest for 3 h, 24 h, 48 h and 72 h, media were aspirated and 0.5 mg/mL MTT (3-(4, 5-dimethylthiazolyl-2)-2, 5-diphenyltetrazolium bromide) (Molecular Probes, USA) was added and incubated for 4 h at 37°C. The formazan crystals formed were dissolved in dimethyl sulfoxide (DMSO) and the absorbance was measured at 570 nm and 650 nm as reference wavelengths using SpectraMax M2e. Cell viability was expressed as relative percentage to the untreated control.

### Gene expressions by Real-Time PCR

RNA was isolated from the treated cells using TRI reagent (Favorgen Biotech Corp, Taiwan) and the cDNA conversion was performed using iScript cDNA synthesis (Biorad, Hercules, USA). The cDNA was used to determine the *VEGF*, *α-SMA* and *NFE2L2 (Nrf2)* gene expressions by Real-Time Polymerase Chain Reaction (qPCR), which was performed with the CFX96 Touch Real-Time detection system (Bio-Rad, USA) using SYBR Green chemistry (Roche, Germany). The qPCR conditions were 95°C for 10 min, followed by 40 cycles of 95°C for 15 s, 60°C for 15 s and 72°C for 20 s. The primer sequences are as given in the supplementary data (S1 Table). The comparative 2^(-ΔΔCt)^ method was used to analyze the results of the genes of interest relative to the internal control gene (*18S rRNA*) [30] and expressed as fold change after normalizing to the untreated control.

### Protein expression by western blotting

The cell lysate was prepared with RIPA (Radioimmunoprecipitation assay) buffer and the total protein estimation was measured using Pierce BCA protein assay kit (Thermo Fisher Scientific, USA) as per the manufacturer’s instructions. The protein resolved in 10% SDS-PAGE was transferred onto Hybond-P PVDF membrane (Amersham Pharmacia Biotech, UK), blocked with 5% BSA (Company, country) and probed with primary antibodies for pNF-κB, NF-κB (Cell Signaling Technology, USA), Bcl-2 (Thermo Fisher Scientific, USA), Bax and β-actin (Santa Cruz Biotechnology, USA). Then the blots were probed with corresponding species-specific HRP-conjugated secondary antibodies (Santa Cruz Biotechnology, Santa Cruz, CA, USA) and developed with Amersham ECL Prime Western Blotting Detection Reagent (GE Healthcare, UK) and the images were captured using FluorChem FC3 gel documentation system (Protein Simple, California, USA). The intensity of the bands were assessed using Image-J software (NIH, Bethesda, USA). β-actin was used as loading control.

### Cytokine estimation

As per the published method of Holtkamp et al, the transwell filters (Costar; 12mm diameter, 0.4 μm pore size) coated with 160 μL of 1:40 dilution of Geltrex-Matrigel (Thermo Fisher Scientific, USA) in DMEM/F-12 medium and air-dried overnight. ARPE-19 cells (1 x 10^5^) were cultured on transwell inserts in low serum media (200 μL per transwell) while the lower compartment had 1.0 mL medium and grown for 19 days. Trans-epithelial electrical resistance (TEER) was measured using EVOM^2^ Epithelial Voltohmmeter (World Precision Instruments, USA) to ensure RPE cell polarity. After subtracting the reading of transwell without cells, the resistance measured in Ω/cm^2^ were > 20 in all the wells indicative of RPE polarity [31]. The polarized nature of the cell was also evaluated based on increase in VEGF elaboration in transwells than cells grown in plates. The cells in transwell were then exposed to the pro-oxidant insults for 24 h. The basal conditioned media was then collected to measure IL-6 and IL-8 secretion by enzyme-linked immunosorbent assay (ELISA) (Peprotech, USA). In another set of experiment cells in transwell exposed to the pro-oxidant insults in the study for 24 h were evaluated for alpha-smooth muscle actin (α-SMA) gene expression as fibroblast marker, to check if transwell grown cells retain epithelial morphology to relate to the cytokine expression and this was compared cells grown in plates and exposed to similar insults using transforming growth factor-beta 1 (TGF-β1) as positive control.

### Chemotactic assay

The ARPE-19 cells (3 x 10^4^) were seeded to 24-well culture plate and allowed to grow till 80% confluency. The THP-1 Macrophage cells (2.5 x 10^5^) was differentiated in transwell inserts of 8 μm pore size (Genetix Biotech Asia, India) separately. ARPE-19 cells were exposed to pro-oxidant insults for 24 h. The Chemotactic assay was performed after 24 h exposure by keeping the transwell inserts with THP-1 Macrophage over the ARPE-19 cells in the 24-well plates containing the conditioned media for 4 h. After 4 h, the THP-1 Macrophage was fixed with 4% paraformaldehyde (Merck, USA) and permeabilized with methanol (Sisco Research Laboratories Pvt. Ltd., India) and stained with Giemsa (Merck, USA). Non-migrated cells were scraped off from the upper surface and migrated cells in the lower surface of the inserts were observed under the bright field in Nikon Eclipse Ts2 microscope (Nikon Instruments Inc., USA) based on the stain at 10X magnification. Five random fields were captured for each insert and the number of migrated cells was counted. Three independent experiments were performed and the data are expressed as relative percentage to control.

### *In vitro* angiogenesis assay

Tube formation assay was performed to check the secretion of pro-angiogenic factors from ARPE-19 using Geltrex-Matrigel. Initially, the 96-well plate was coated with Geltrex-Matrigel (35 μL/well) carefully without air bubbles and allowed to polymerize for 30 min at 37°C. Rhesus monkey Choroidal-retinal endothelial cells (RF/6A, #CRL1780, American Tissue Culture Collection) was subjected to low serum media for overnight and added at a density of 1.5 x 10^4^ cells/well and mixed with 100 μL conditioned media of ARPE-19 treated with pro-oxidant conditions into 96-well plate coated with Geltrex-Matrigel. Vascular endothelial growth factor (VEGF; 10 ng/mL) was used as positive control. Tube formation on the gel surface was documented with Axio Observer Z.1 microscope (Carl Zeiss, Germany) after 4 h. Three random fields were captured in each well and the tube length was measured using the ImageJ software [32].

### AMD patient recruitment for assessing pro-inflammatory cytokines in serum

As part of a prospective study in 2010–2013 in a tertiary eye care centre in south India, 23 AMD patients (Mean age: 68.8 ± 2.1 years, M/F: 17/6) and 23 healthy control subjects (Mean age: 53.7 ± 1.8 years, M/F: 14/9) were recruited in this study. The study was conducted in adherence to the principles of the Helsinki declaration and approved by the Ethics Sub-Committee of Vision Research Foundation, Sankara Nethralaya (Study Code: 149-2009-P, dated 29.08.2009). A written informed consent was obtained from the study participants. Detailed ophthalmic and medical evaluation was done and subjects with history of Diabetes Mellitus (DM), renal dysfunction, hepatic disease, or inflammatory diseases and presence of retinal diseases other than AMD such as high myopia, retinal dystrophies, central serous retinopathy, vein occlusion, diabetic retinopathy, and uveitis or similar outer retinal diseases which has been present prior to the age of 50 years were excluded. AMD diagnosis was based on the Age-Related Eye Disease Study (AREDS) guidelines [33]. The laboratory investigations of biochemical tests done and the clinical data are given in the supplementary data (S2 Table and S1 File). Serum IL-6 and IL-8 levels were estimated in AMD patients and control subjects by ELISA.

### Statistics

All data are expressed as Mean ± SEM of 3 independent experiments done not less than duplicates unless indicated. Statistical significance was assessed by Student’s *t*-test. *p < 0*.*05* was considered statistically significant.

## Results

### ROS generation after exposure to oxLDL and Hcy

**Fig 1:**
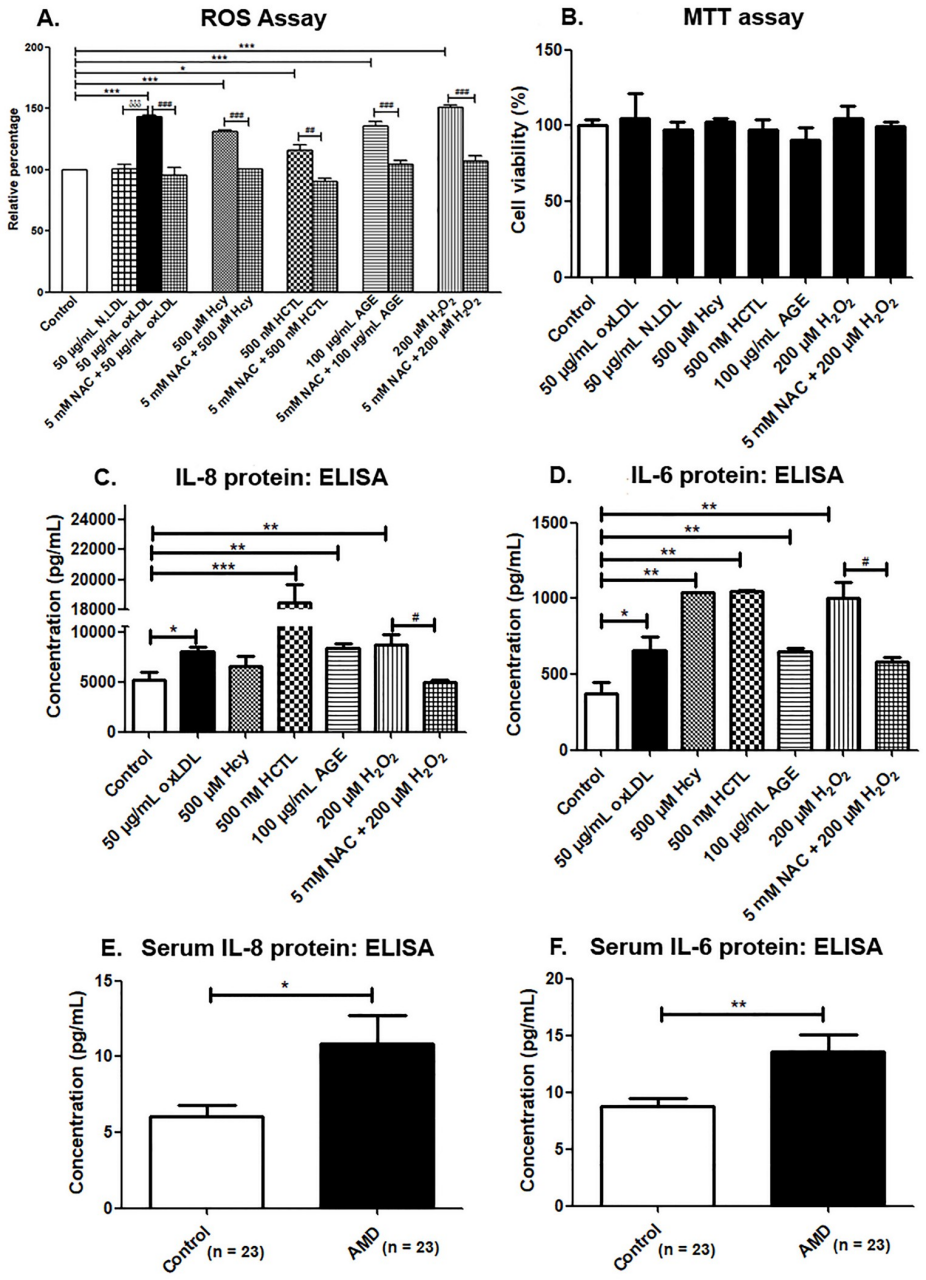
ROS assay and pro-inflammatory cytokine levels. (A) ROS assay in ARPE-19 cells by DCFH-DA method, (B) MTT assay for cell viability in ARPE-19 cells, (C) IL-8 and (D) IL-6 estimation in basal conditioned media of ARPE-19 cells exposed to pro-oxidants conditions for 24 h by ELISA, (E) Serum IL-8 levels and (F) Serum IL-6 levels. Values are expressed as Mean ± SEM. *^;*#*^*p < 0*.*05*, **^,*##*^*p < 0*.*01*, ***;^*###;δδδ*^*p < 0*.*001*, considered as significant. *Control vs pro-oxidants, ^*#*^pro-oxidants vs NAC, ^*δ*^oxLDL vs N.LDL.

The oxidative capability of the metabolites was assessed by intracellular ROS and MDA levels in ARPE-19 cells. A significant increase in the ROS level was observed in the ARPE-19 cells exposed to oxLDL compared to untreated and N.LDL treated cells (*p < 0*.*001*). Hcy, AGE (*p < 0*.*001*) and HCTL (*p < 0*.*05*) also promoted ROS generation and thus acted as significant pro-oxidants. The anti-oxidant, NAC significantly reduced the ROS levels induced by the metabolites (Fig 1A). The intracellular MDA was significantly increased by the metabolites studied. This was lowered by the NAC treatment, thus showing the oxidative stress in ARPE-19 cells caused by the metabolites (S1 Fig).

### Cell viability assay

MTT assay was performed to find out the cytotoxic effect of the compounds studied in ARPE-19 cells. Cells were treated with 50 μg/mL oxLDL, 50 μg/mL N.LDL, 500 μM Hcy, 500 nM HCTL, 100 μg/mL AGE and 200 μM H_2_O_2_ for various time points. The cells were viable under all the treatment conditions and no significant cell death was observed at 24 h (Fig 1B). This was further substantiated by the trypan blue assay that showed less than 10% cell death with pro-oxidants treatment at both 24 h and 72 h, which was not statistically significant (S2 Fig). However, there was around 20 percent reduction in the MTT assay with the metabolites at 72 h, which can be attributed to the loss of mitochondrial activity (S3 Fig). Thus the metabolites caused no significant cell death in spite of their pro-oxidant effect in ARPE-19 cells.

### Pro-inflammatory cytokines are secreted by APRE-19 cells on treatment with oxLDL, Hcy and AGE

Increased oxidative stress can trigger the inflammatory response in RPE. Therefore, IL-6 and IL-8 were evaluated amongst the cytokines reportedly associated with AMD. IL-8 secretion by ARPE-19 cells was significantly elevated on treatment with oxLDL (*p < 0*.*05*), AGE and H_2_O_2_ (*p < 0*.*01*). However, HCTL showed maximal induction (*p < 0*.*001*), while Hcy treatment did not alter it significantly (Fig 1C). IL-6 secretion by ARPE-19 cells was significantly elevated with oxLDL (*p < 0*.*05*) and AGE treatment (*p < 0*.*01*). Hcy and HCTL treatments showed maximal IL-6 release similar to that of H_2_O_2_ treatment (*p < 0*.*01*) (Fig 1D). Thus, the pro-oxidants studied promoted inflammatory response in ARPE-19 cells.

When these specific inflammatory markers were assessed at the systemic level, the serum IL-8 and IL-6 levels were found to be significantly elevated in the AMD patients (10.48 ± 1.79 pg/mL and 12.97 ± 1.46 pg/mL, respectively) compared to control subjects (6.07 ± 0.76 pg/mL and 8.76 ± 0.62 pg/mL, correspondingly) (Fig 1E and 1F). Thus, inflammation is observed at the systemic level in AMD as seen by the cytokine markers studied. S2 Table shows the systemic biochemical details of the AMD patients, wherein significantly altered plasma lipid profile was observed in terms of increased total cholesterol, triglycerides and very low-density lipoprotein (VLDL).

Cells grown in transwell inserts were evaluated for their epithelial nature. The gene expression of the fibroblastic marker, *α-SMA* was not increased in the cells treated with the pro-oxidants at the concentration and time point studied (S4 Fig). Hence, the cytokines measured in transwell are from the polarized and epithelial form of RPE cells. However, a significant increase in *α-SMA* was observed in cells cultured in tissue culture plates in response to all the pro-oxidants, similar to the insult of TGF-β1 at 5 ng/mL (positive control).

### Increased VEGF secretion with oxLDL, Hcy and AGE treatment

**Fig 2:**
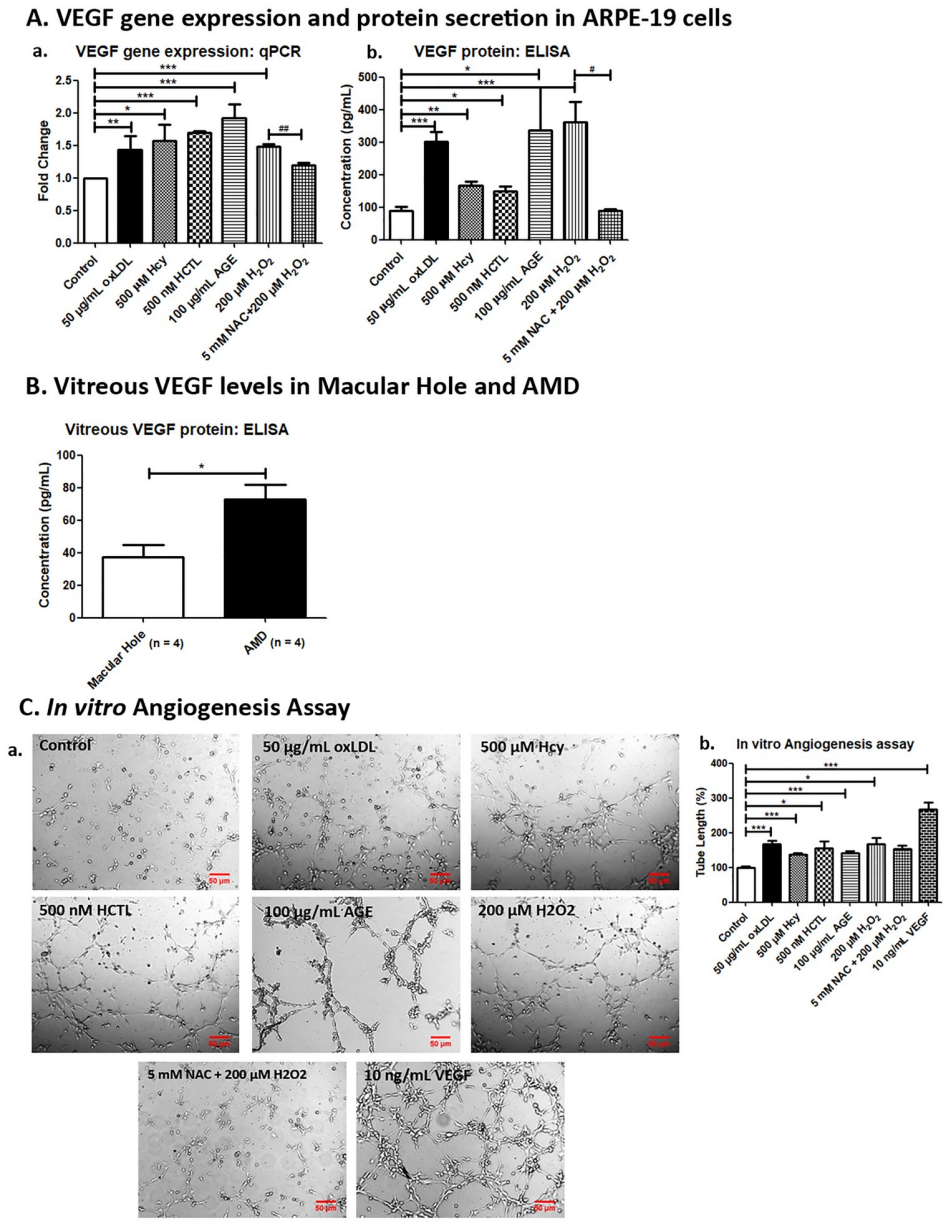
Pro-angiogenic effect of the pro-oxidants in ARPE-19 cells. (A) VEGF gene expression and protein secretion in ARPE-19 cells. (a) *VEGF* gene expression was quantified by qPCR in ARPE-19 cells exposed to pro-oxidant conditions for 24 h, (b) VEGF secretion was estimated by ELISA in conditioned media of ARPE-19 cells exposed to pro-oxidant conditions for 24 h. (B) Vitreous VEGF levels in Macular Hole and AMD. VEGF protein levels in vitreous of idiopathic Macular Hole subjects and AMD patients were estimated by ELISA. (C) *In vitro* Angiogenesis Assay. (a) RF/6A cells were treated with conditioned media of ARPE-19 exposed to pro-oxidant conditions and 10 ng/mL VEGF for 4 h and images were captured using Axio Observer Z.1 microscope at 5X magnification, (b) Bar graph represents the quantification of tube length measured using ImageJ software and expressed as relative percentage to control. The data are represented as Mean ± SEM. *^;*#*^*p < 0*.*05*, **^;*##*^*p < 0*.*01*, ***p < 0.*001*, considered as significant. *Control vs pro-oxidants, ^*#*^H_2_O_2_ vs NAC.

Oxidative stress and chronic inflammation in the RPE and retina are reported to be associated with choroidal neovascularization in AMD. Hence, we measured the VEGF expression in ARPE-19 exposed to pro-oxidant metabolites. Treatment with oxLDL, Hcy, HCTL and AGE showed a significant increase in the *VEGF* mRNA and protein expression at 24 h (Fig 2A). The VEGF levels in vitreous of AMD patients (83.98 ± 20.31 pg/mL) was significantly higher compared to idiopathic Macular Hole subjects (30.15 ± 7.78 pg/mL) (Fig 2B). These results showed that these metabolites pave way for the wet AMD.

### Angiogenesis is promoted by oxLDL, Hcy and AGE

As the VEGF secretion increased by ARPE-19 under oxidative stress, the effect of conditioned media of ARPE-19 cells exposed to the pro-oxidants was assessed for pro-angiogenic effect by *in vitro* angiogenesis assay in RF6A endothelial cells. There was a significant increase in tube formation on exposures to oxLDL, Hcy, HCTL, AGE and H_2_O_2_ by 67.7%, 36.9%, 56.2%, 41.9% and 67.2%, respectively compared to control. VEGF treatment used as a positive control showed an increase by 167.5% and NAC treatment decreased this by 15% (Fig 2C). This shows that oxLDL, Hcy, HCTL and AGE promote angiogenesis which is characteristic of wet AMD.

### Oxidative stress promotes chemotaxis of macrophages

**Fig 3:**
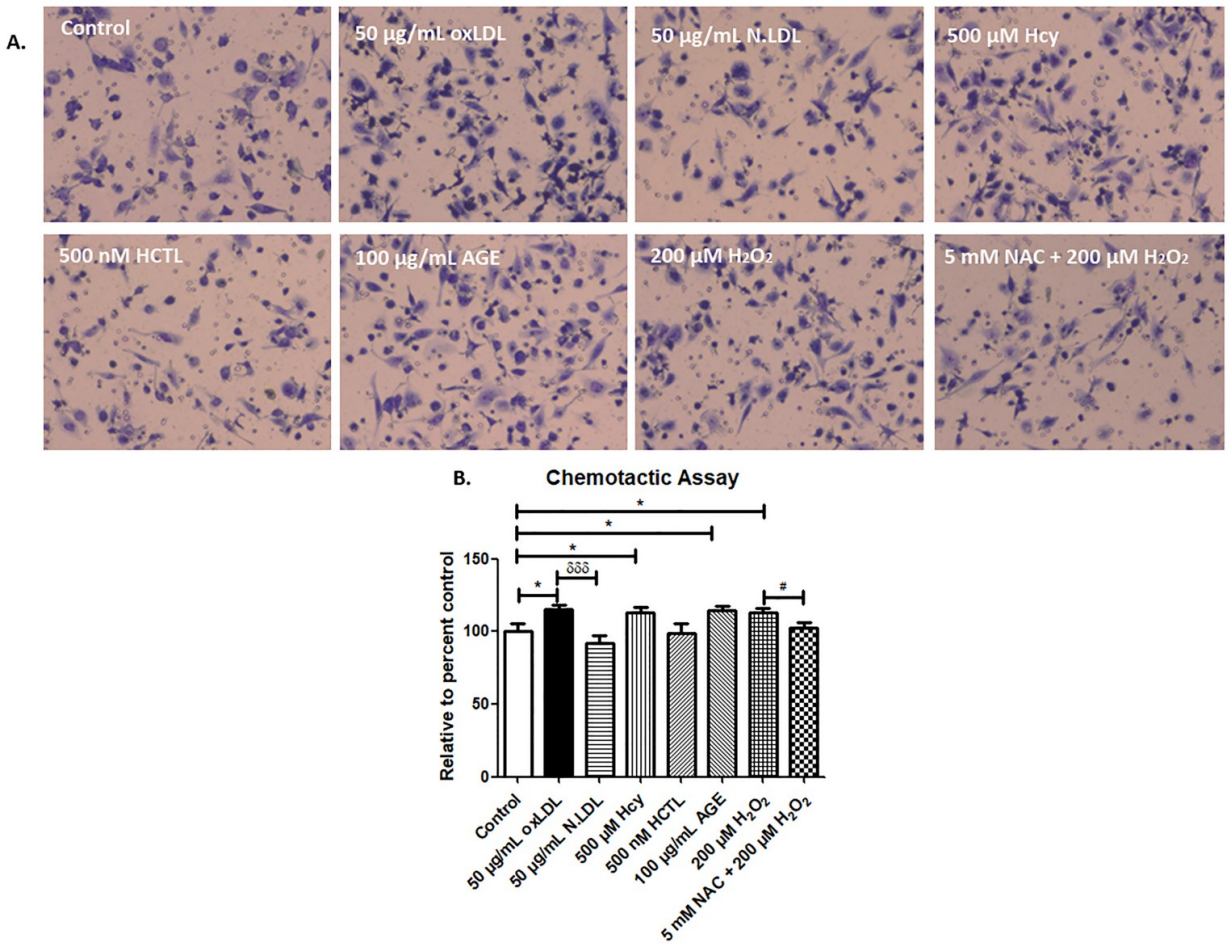
Chemotactic assay of THP-1 macrophages. (A) Transwell migration of THP-1 macrophage in response to the cytokine secretion of ARPE-19 cells exposed to pro-oxidant conditions for 24 h was assessed after 4 h incubation. The migrated macrophages were stained with Giemsa stain and five random fields were captured per well with Nikon Eclipse Ts2 microscope at 10X magnification. (B) Bar chart showing the number of migrated macrophages, represented as relative percentage to control. Values are expressed as Mean ± SEM, n = 3. *p < 0.*05*, ^*δδδ*^*p < 0*.*001*, considered as significant. *Control vs pro-oxidants, ^*#*^H_2_O_2_ vs NAC, ^*δ*^oxLDL vs N.LDL.

Macrophage infiltration is one of the key events in the pathogenesis for choroidal neovascularization in wet AMD. Hence, we performed the transwell migration assay to assess the macrophage infiltration in response to the secretion of ARPE-19 cells exposed to pro-oxidant conditions. This assay showed that there was a significant increase in the migration of macrophages in response to oxLDL, Hcy, AGE by 15.3%, 13.2% and 14.4%, respectively compared to control. NAC treatment showed an 11% decrease compared to H_2_O_2_-alone treatment (Fig 3A and 3B). This result indicates that the oxidative stress induced by these metabolites in ARPE-19 cells promote secretion of pro-inflammatory cytokines, which in turn causes macrophage infiltration.

### Oxidative stress promotes pro-apoptotic changes via NF-κB signaling

**Fig 4:**
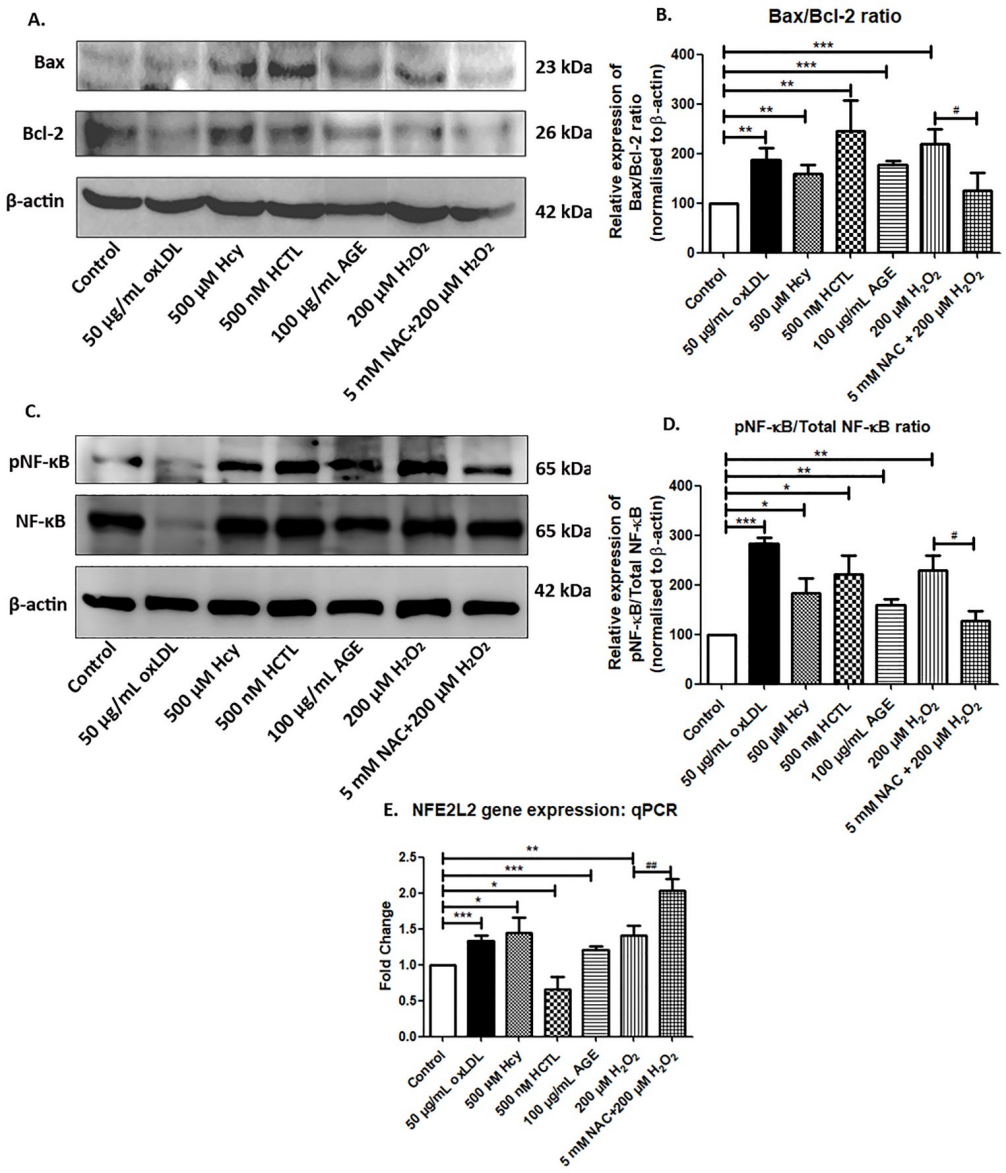
Pro-apoptotic changes in ARPE-19 cells. (A) Bax and Bcl-2 protein expression in ARPE-19 under the pro-oxidants conditions treated for 24 h was assessed by western blot. (B) Densitogram analysis of Bax/Bcl-2 ratio normalized to control. (C) pNF-κB and NF-κB protein expression in ARPE-19 under the pro-oxidants conditions treated for 24 h was assessed by western blot. (D) Densitogram analysis of pNF-κB/NF-κB ratio normalized to control. (E) *NFE2L2* gene expression was quantified by qPCR in ARPE-19 cells exposed to pro-oxidant conditions for 24 h. Values are expressed as Mean ± SEM, n = 3. *^;*#*^*p < 0*.*05*, **^,*##*^*p < 0*.*01*, ***p < 0.*001*, considered as significant. *Control vs pro-oxidants, ^*#*^H_2_O_2_ vs NAC.

To investigate whether the pro-oxidants induce pro-apoptotic changes in ARPE-19 cells, the protein expression of Bcl-2 (anti-apoptotic) and Bax (pro-apoptotic), as well as the activation of the pro-apoptotic transcription NF-κB protein by its phosphorylation were evaluated. There was a significant increase in the Bax/Bcl-2 ratio with the pro-oxidant treatments (Fig 4A and 4B) along with significant activation of NF-κB in ARPE-19 cells (Fig 4C and 4D). Further, *NFE2L2*, transcription factor which is known to be increased in oxidative stress as a defence was evaluated and found to be increased by the pro-oxidants treatment (Fig 4E). With HCTL, *NFE2L2* expression was significantly lower though NF-κB was increased. However, net oxidative stress was observed in HCTL treament with respect to ROS and MDA levels. Interestingly, NAC treatment further increased the *NFE2L2* expression in RPE unlike in other cells reported [34]. Annexin V staining supported the pro-apoptotic changes of ARPE-19 cells under the pro-oxidant conditions (S5 Fig).

## Discussion

Oxidative stress is one of the major contributors to the pathogenesis of AMD. Many systemic pro-oxidant metabolites are formed during oxidative stress pathologies such as the lipid-derived reactive aldehydes, proteins / amino acid oxidative markers such as nitrotyrosine, nucleic acid oxidation product such as 8-hydroxy deoxyguanosine, the prostaglandin derived F2-isoprostanes and the redox imbalance marker, Hcy. Association of such metabolites with AMD pathology has been reported widely [13,35–38].

This study focussed on oxLDL which is not extensively studied in RPE in the context of AMD. In our previous study in AMD, we found that serum oxLDL was significantly elevated in AMD patients. The study showed that compared to native LDL, oxLDL increases ROS in ARPE-19 cells. The oxLDL level is reported as a biomarker of many oxidative stress involving pathologies such as atherosclerosis, cerebral infarction and AMD as well [39]. Formation of lipid-protein adducts such as MDA-lysine and 4-HNE-lysine on apolipoprotein B can induce ROS formation [40]. ROS generation as well as metabolism of oxLDL, can promote RPE cell dysfunction due to pathological stress response contributing to AMD pathology [41].

Though controversial, Hcy at the systemic level has been associated with AMD [42]. Our previous study showed that high plasma Hcy and HCTL levels were not significantly different from that of the age-matched controls [9]. However, recent studies reveal that it can potentially cause RPE dysfunction [15,43]. This study explored to delineate their effect localized to RPE. Accordingly, Hcy and HCTL increased the ROS levels in ARPE-19 cells acting as pro-oxidants. Hcy can undergo cyclization to form HCTL which brings about N-homocysteinylation of proteins including that of LDL. These Hcy-*N*-proteins are capable of initiating oxidative damages [44,45]. HCTL, released from homocysteinylated LDL causes oxidation of cholesterol [46]. Hcy and HCTL also mediate MDA accumulation in the microvascular endothelial cells as reported by us earlier [47]. More studies are warranted to delineate its role in AMD pathology at the local environment rather than relating to systemic hyperhomocysteinemia.

Accumulation of AGE with aging is associated with drusens and is known to promote receptor for AGE (RAGE) mediated neovascularization and apoptosis in AMD [23]. AGE levels increase with oxidative stress and the accumulated AGE, in turn, can increase ROS levels apart from glycating the proteins [48]. Methylglyoxal promotes cell death through ROS generation in ARPE-19 cells [49]. The study shows the comparison of the metabolites including that of the AGE treatment that can promote AMD pathology at the level of oxidative stress, inflammation, apoptosis and pro-angiogenic effect.

Increase in IL-6 and IL-8 secretions were observed in ARPE-19 cells on treatment with the pro-oxidants studied. Evidences based on histological studies has shown chronic inflammation in RPE and choroid with infiltration of lymphocytes and macrophages in AMD eyes [50]. Interestingly, serum pro-inflammatory cytokines, IL-6 and IL-8 were found to be high in AMD patients. Thus, there is a systemic inflammation as well as the inflammatory component of the RPE environment involved in AMD pathology. While a recent report states that no systemic changes in serum or aqueous humour in any of the cytokine was observed in AMD, a panel of altered cytokines has also been reported in AMD [51,52]. While the method of detection seems to be a point of concern, more studies in larger groups are warranted to understand the local versus systemic markers of the inflammation status in AMD.

Increased VEGF expression observed on pro-oxidant treatment in ARPE-19 reveals that the local environment of RPE is susceptible to oxidative stress. *In vitro* angiogenesis assay revealed factors secreted by RPE in response to the intracellular ROS generated promote neovascularization. There was a significant increase in VEGF levels in vitreous of AMD patients compared to idiopathic Macular Hole subjects. However, it is shown in a small sample size which is a limitation in the study. Inflammasome activation by lipids is reported both at the systemic level and in the RPE local environment in AMD [53]. Moreover, oxLDL, Hcy and AGE induce VEGF secretion also in macrophages [54–57]. The role of macrophages in the pathological neovascularization remains elusive. While Apte et al., 2006 showed macrophages are protective in limiting neovascular process as seen in laser-induced CNV mice model, earlier studies had shown that depletion of macrophages blocked neovascularization in mouse models [58,59]. However, CNV is associated with specific M2 polarized macrophages [60]. Monocyte/macrophage recruitment in response to cytokine secretion is reported in cell models [61]. We found THP-1 macrophage chemotaxis in response to cellular supernatant by the ARPE-19 exposed to pro-oxidants. Thus, the macrophage infiltration was mediated by cytokine elaborated by the ARPE-19 exposed to these pro-oxidant metabolites.

Oxidative stress induces pro-apoptotic changes in various cells [62–64]. The oxLDL induces apoptosis of RPE through activation of ERK-Bax/Bcl-2 signaling pathways [65]. We found that the pro-oxidants oxLDL, Hcy, HCTL and AGE increased the Bax to Bcl-2 ratio and activation of NF-κB, showing pro-apoptotic changes in ARPE-19. Activation of NF-κB inducing apoptosis in response to various stimuli such as oxLDL, Hcy and AGE has been reported earlier [64,66,67]. Interestingly, HCTL seems to be more deleterious as there is a significant decrease in *NFE2L2* expression which is supported by the observation that IL-8 secretion is maximal in HCTL treatment. Lowering of *NFE2L2* expression reportedly promotes inflammation [68]. NAC treatment in RPE unlike in other cells did not suppress *NFE2L2* expression and in fact further increased the expression over that of H_2_O_2_ treatment. This is probably characteristic of RPE and needs further studies. Earlier we had reported that PON2, an antioxidant increased by H_2_O_2_ treatment was further increased with NAC treatment in ARPE-19 cells [69].

Oxidative stress induces epithelial to mesenchymal transition that leads to functional deficiencies of RPE in AMD [70]. In this study, only the cytokine release and chemotactic response were done in transwell grown polarized cells wherein the cells are epithelial in nature. However, the rest were done in plate culture where RPE exhibits mesenchymal change with the pro-oxidants which is a study limitation. There are both epithelial and mesenchymal RPE in AMD and more studies are required in the *in vivo* condition to evaluate EMT with disease progression.

## Conclusion

The study showed that the metabolites studied induce intracellular ROS generation in ARPE-19 cells promoting secretion of pro-inflammatory cytokines. The cytokines recruit macrophages at the site of RPE and induce VEGF secretion promoting neovascularization. Moreover, these pro-oxidant metabolites induce pro-apoptotic changes in RPE through NF-κB signaling.

## Supporting information

S1 Fig MDA assay in ARPE-19 cells. ARPE-19 cells were exposed to metabolites namely 50 μg/mL oxLDL, 500 μM Hcy, 500 nM HCTL, 100 μg/mL AGE, 200 μM H_2_O_2_ for 24 hours and with or without pre-treatment with 5 mM NAC for 1 hour. After exposure, the cells were lysed with 0.5% Triton X 100 and measured the MDA formed by MDA assay. The data are represented as Mean ± SEM. *^;*#*^*p < 0*.*05*, **^,*##*^*p < 0*.*01*, considered as significant. *Control vs pro-oxidants; ^*#*^pro-oxidants vs NAC.
(TIF)

S2 Fig Trypan Blue assay for cell viability in ARPE-19 cells. ARPE-19 cells were exposed to pro-oxidants for 24 h (A) and 72h (B). The cell viability was measured by Trypan Blue assay. At the end of the exposure, the cell suspension after trypsinization was mixed with 0.4% trypan blue solution (1:1) and counted the stained (dead) and unstained (viable) cells in a hemocytometer. The data are expressed as cell viability (%) relative to control and is a mean of three independent experiments (Mean ± SEM).
(TIF)

S3 Fig MTT assay in ARPE-19 cells. MTT assay was performed at different time points such as 3, 24, 48, and 72 h with the exposures of 50 μg/mL oxLDL, 500 μM Hcy, 500 nM HCTL, 100 μg/mL AGE, 200 μM H_2_O_2_ and 5 mM NAC pre-treatment with 200 μM H_2_O_2_. Nearly 20% fall in the mitochondrial activity was observed with the metabolites treatment at 72 h with all the exposures except 100 μg/mL AGE (< 5%).
(TIF)

S4 Fig *α-SMA* gene expression in ARPE-19. *α-SMA* gene expression was quantified by qPCR in ARPE-19 cells exposed to pro-oxidant conditions for 24 h. (A) ARPE-19 cells grown on 12-well transwell inserts, (B) ARPE-19 cells grown in 12-well tissue culture plate. The fold change (Y-axis) is calculated after normalizing to untreated control as detailed in the method section 2.7. The data are represented as Mean ± SEM. *p < 0.*05*, ***p < 0.*001*, considered as significant. *Control vs pro-oxidants.
(TIF)

S5 Fig Annexin V staining showing apoptotic changes in ARPE-19 cells. ARPE-19 cells were exposed to pro-oxidants for 24 h and apoptotic changes were observed by Annexin V staining. FITC labelled Annexin V was indicated by green fluorescence. Image magnification, 40X.
(TIF)

S1 Table List of primers used for Real-Time PCR. (DOC)

S2 Table Biochemical parameters in AMD and control. Data are expressed as Mean ± SEM; M: Male; F: Female; HDL: High-density lipoprotein; LDL: Low-density lipoprotein; VLDL: Very low-density lipoprotein; TC: Total cholesterol. NS: Not significant.
(DOC)

S1 File Clinical data of participants recruited in the study. (XLS)
