# Supplementary figures and images for: Oxidized LDL, homocysteine, homocysteine thiolactone and advanced glycation end products act as pro-oxidant metabolites inducing cytokine release, macrophage infiltration and pro-angiogenic effect in ARPE-19 cells
Source: PLoS One. 2019 May 14;14(5):e0216899. doi: 10.1371/journal.pone.0216899 (PMC6516731; doi:10.1371/journal.pone.0216899)

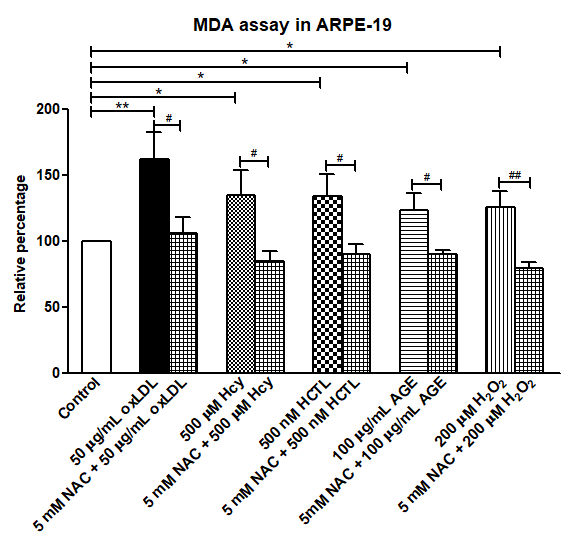

Supplement: S1 Fig — ARPE-19 cells were exposed to metabolites namely 50 μg/mL oxLDL, 500 μM Hcy, 500 nM HCTL, 100 μg/mL AGE, 200 μM H2O2 for 24 hours and with or without pre-treatment with 5 mM NAC for 1 hour. After exposure, the cells were lysed with 0.5% Triton X 100 and measured the MDA formed by MDA assay. The data are represented as Mean ± SEM. *;#p < 0.05, **,##p < 0.01, considered as significant. *Control vs pro-oxidants; #pro-oxidants vs NAC. (TIF) [file pone.0216899.s001.tif]

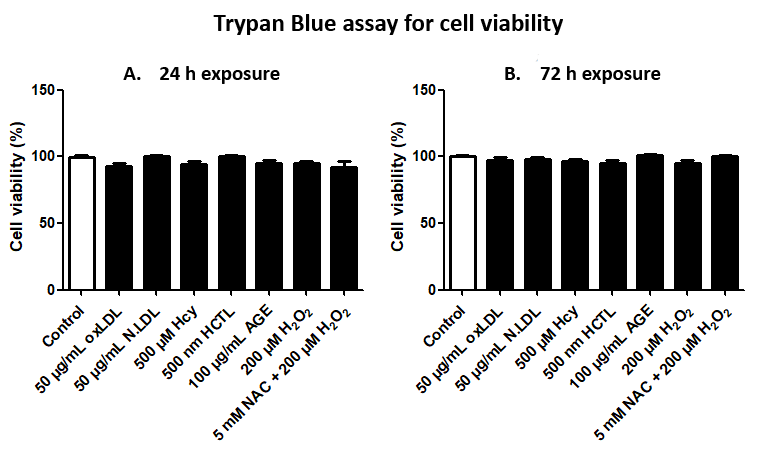

Supplement: S2 Fig — ARPE-19 cells were exposed to pro-oxidants for 24 h (A) and 72h (B). The cell viability was measured by Trypan Blue assay. At the end of the exposure, the cell suspension after trypsinization was mixed with 0.4% trypan blue solution (1:1) and counted the stained (dead) and unstained (viable) cells in a hemocytometer. The data are expressed as cell viability (%) relative to control and is a mean of three independent experiments (Mean ± SEM). (TIF) [file pone.0216899.s002.tif]

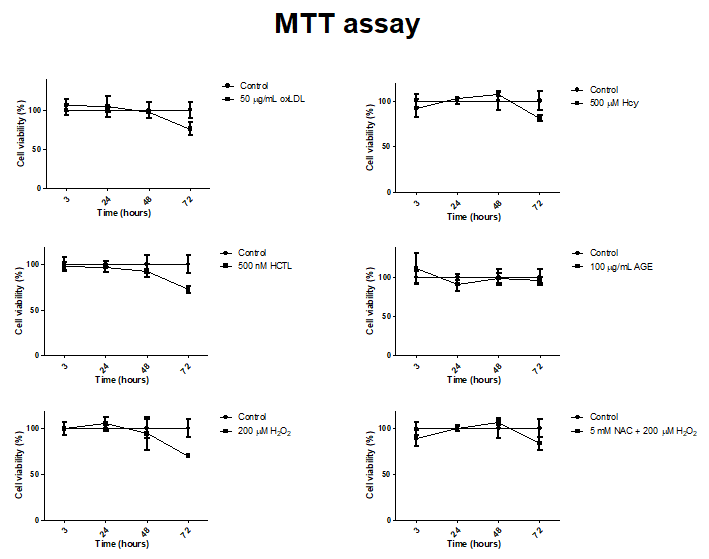

Supplement: S3 Fig — MTT assay was performed at different time points such as 3, 24, 48, and 72 h with the exposures of 50 μg/mL oxLDL, 500 μM Hcy, 500 nM HCTL, 100 μg/mL AGE, 200 μM H2O2 and 5 mM NAC pre-treatment with 200 μM H2O2. Nearly 20% fall in the mitochondrial activity was observed with the metabolites treatment at 72 h with all the exposures except 100 μg/mL AGE (< 5%). (TIF) [file pone.0216899.s003.tif]

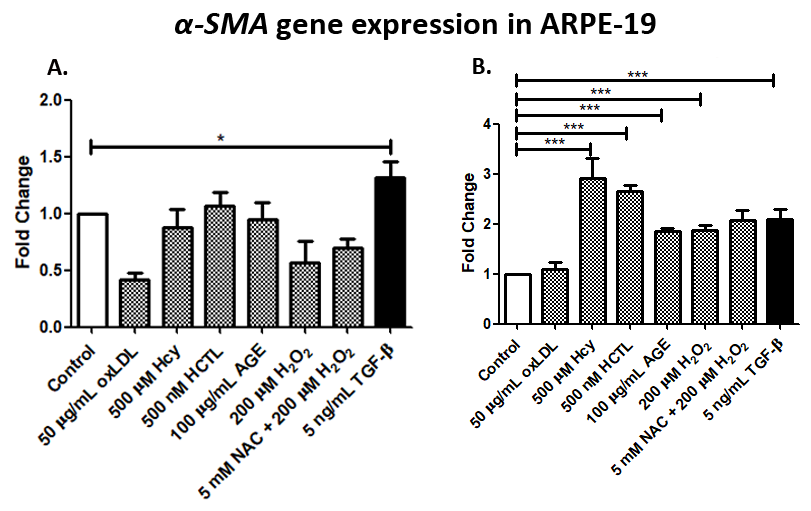

Supplement: S4 Fig — α-SMA gene expression was quantified by qPCR in ARPE-19 cells exposed to pro-oxidant conditions for 24 h. (A) ARPE-19 cells grown on 12-well transwell inserts, (B) ARPE-19 cells grown in 12-well tissue culture plate. The fold change (Y-axis) is calculated after normalizing to untreated control as detailed in the method section 2.7. The data are represented as Mean ± SEM. *p < 0.05, ***p < 0.001, considered as significant. *Control vs pro-oxidants. (TIF) [file pone.0216899.s004.tif]

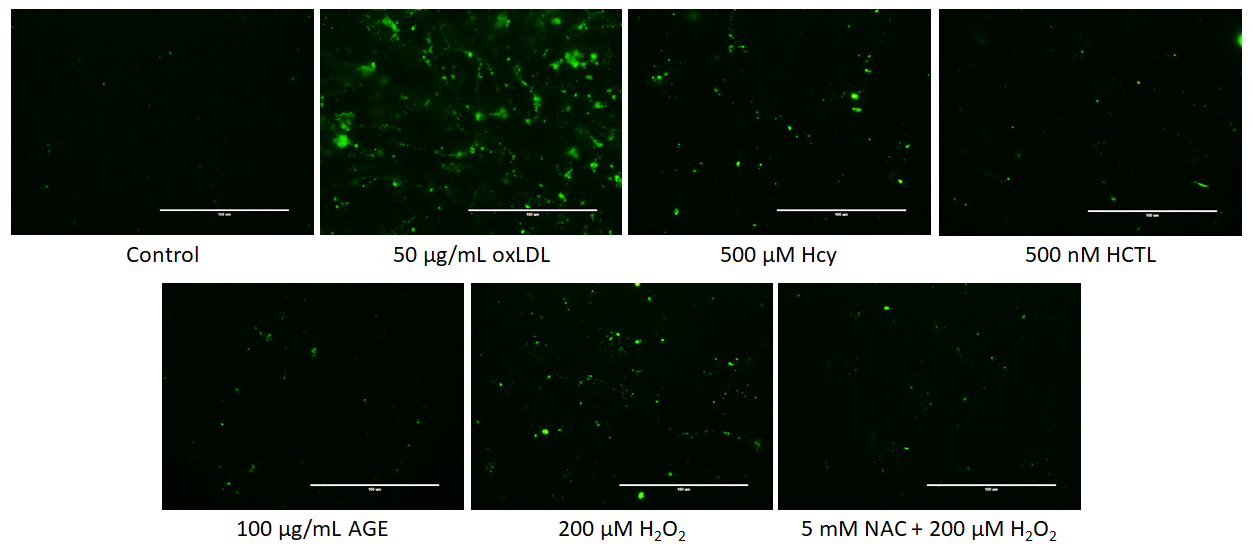

Supplement: S5 Fig — ARPE-19 cells were exposed to pro-oxidants for 24 h and apoptotic changes were observed by Annexin V staining. FITC labelled Annexin V was indicated by green fluorescence. Image magnification, 40X. (TIF) [file pone.0216899.s005.tif]
