# Supplementary material for: Oxidized LDL, homocysteine, homocysteine thiolactone and advanced glycation end products act as pro-oxidant metabolites inducing cytokine release, macrophage infiltration and pro-angiogenic effect in ARPE-19 cells
Source: PLoS One. 2019 May 14;14(5):e0216899. doi: 10.1371/journal.pone.0216899 (PMC6516731; doi:10.1371/journal.pone.0216899)
Supplement: S1 Table — (DOC) [file pone.0216899.s006.doc]

**S1 Table. List of primers used for Real-Time PCR**

| **S.No.** | **Gene** | **Forward primer sequence** | **Reverse primer sequence** | **Product Size (bp)** |
| --- | --- | --- | --- | --- |
| 1 | VEGF | 5’-GTCCAACTTCTGGGCTGTTCTC-3’ | 5’-CCCCTCTCCTCTTCCTTCTCTT-3’ | 152 |
| 2 | α-SMA | 5’-GGCTGTTTTCCCATCCATTGT-3’ | 5’-TCTTTTGCTCTGTGCTTCGT-3’ | 103 |
| 3 | NFE2L2 | 5’-CGGTATGCAACAGGACATTG-3’ | 5’-GTTTGGCTTCTGGACTTGGA-3’ | 125 |
