# Supplementary material for: Oxidized LDL, homocysteine, homocysteine thiolactone and advanced glycation end products act as pro-oxidant metabolites inducing cytokine release, macrophage infiltration and pro-angiogenic effect in ARPE-19 cells
Source: PLoS One. 2019 May 14;14(5):e0216899. doi: 10.1371/journal.pone.0216899 (PMC6516731; doi:10.1371/journal.pone.0216899)
Supplement: S2 Table — Data are expressed as Mean ± SEM; M: Male; F: Female; HDL: High-density lipoprotein; LDL: Low-density lipoprotein; VLDL: Very low-density lipoprotein; TC: Total cholesterol. NS: Not significant. (DOC) [file pone.0216899.s007.doc]

**S2 Table. Biochemical Parameters in AMD and Control**

| **Biochemical Parameters** | **Control (Mean ± SEM)** | **AMD (Mean ± SEM)** | ***p value*** |
| --- | --- | --- | --- |
| Sample Size (n) | 23 (17M, 6F) | 23 (14M, 9F) | NS |
| Blood Glucose level (mg/dL) | 97.48±1.60 | 97.83±1.43 | NS |
| Total Cholesterol (mg/dL) | 166.60±4.22 | 193.52±8.40 | ***<0.01*** |
| Triglycerides (mg/dL) | 74.70±6.19 | 165.26±19.54 | ***<0.001*** |
| HDL (mg/dL) | 40.61±1.80 | 43.00±2.50 | NS |
| LDL (mg/dL) | 111.02±4.20 | 117.47±8.28 | NS |
| VLDL (mg/dL) | 14.94±1.24 | 33.05±3.91 | ***<0.001*** |
| TC/HDL ratio | 4.24±0.23 | 4.74±0.31 | NS |
| Total protein (g/dL) | 7.39±0.08 | 7.29±0.13 | NS |

Data are expressed as Mean ± SEM; M: Male; F: Female; HDL: High density lipoprotein; LDL: Low density lipoprotein; VLDL: Very low density lipoprotein; TC: Total cholesterol. NS: Not significant.
